# Quantitative analysis and comparison of 3D morphology between viable and apoptotic MCF-7 breast cancer cells and characterization of nuclear fragmentation

**DOI:** 10.1371/journal.pone.0184726

**Published:** 2017-09-08

**Authors:** Yuhua Wen, Zhan Chen, Jianfen Lu, Elizabeth Ables, Jean-Luc Scemama, Li V. Yang, Jun Q. Lu, Xin-Hua Hu

**Affiliations:** 1 Institute for Advanced Optics, Hunan Institute of Science and Technology, Yueyang, Hunan, China; 2 School of Physics, Hunan Institute of Science and Technology, Yueyang, Hunan, China; 3 Department of Physics, East Carolina University, Greenville, North Carolina, United States of America; 4 Department of Biology, East Carolina University, Greenville, North Carolina, United States of America; 5 Department of Internal Medicine, Brody School of Medicine, East Carolina University, Greenville, North Carolina, United States of America; University of South Alabama Mitchell Cancer Institute, UNITED STATES

## Abstract

Morphological changes in apoptotic cells provide essential markers for defining and detection of apoptosis as a fundamental mechanism of cell death. Among these changes, the nuclear fragmentation and condensation have been regarded as the important markers but quantitative characterization of these changes is yet to be achieved. We have acquired confocal image stacks of 206 viable and apoptotic MCF-7 cells stained by three fluorescent dyes. Three-dimensional (3D) parameters were extracted to quantify and compare their differences in morphology. To analyze nuclear fragmentation, a new method has been developed to determine clustering of nuclear voxels in the reconstructed cells due to fluorescence intensity changes in nuclei of apoptotic cells. The results of these studies reveal that the 3D morphological changes in cytoplasm and nuclear membranes in apoptotic cells provide sensitive targets for label-free detection and staging of apoptosis. Furthermore, the clustering analysis and morphological data on nuclear fragmentation are highly useful for derivation of optical cell models and simulation of diffraction images to investigate light scattering by early apoptotic cells, which can lead to future development of label-free and rapid methods of apoptosis assay based on cell morphology.

## Introduction

Apoptosis is an important mechanism of cell death and its research has wide implications in life science and clinical applications such as drug development for treatment of cancers and other diseases [1, 2]. Apoptotic cells of different phenotypes present strikingly similar changes in three dimensional (3D) morphology that define apoptosis as a unique cell death mode. These changes are consequences of common molecular signaling pathways and can serve as markers for detection and staging of apoptosis [3]. Significant progress has been made in understanding of the molecular pathways and discovery of characteristic signatures for detection by fluorescence assays [4]. Still, use of fluorescence reagents in conventional apoptosis assays has drawbacks that includes additional cell death by fluorophore cytotoxicity, non-uniformity in molecular affinity and emission instability of the reagents among measured cells [5], not to mention the preparation time and reagent cost. Consequently, development of morphology based and label-free methods is very attractive for their potentials to achieve direct or morphology-based and nearly disturbance-free detection of apoptosis. For example, a method of polarization diffraction imaging flow cytometry (p-DIFC) has been shown to have the capability for acquisition of images whose diffraction patterns correlate highly to the 3D morphology of imaged cells by recording spatial distribution of coherent light scatter [6–11]. To establish the p-DIFC method as a new tool for apoptosis assay, it is necessary to quantify 3D morphological changes in apoptotic cells and especially in their nuclei that affect the spatial distribution of scattered light measured as diffraction image. Numerous studies have been reported to visualize 3D structures of cells but investigations of 3D morphology on intracellular organelles are very limited [12–15]. Clear understanding of 3D morphology about apoptotic cells and the differences from viable ones is also required for accurate simulations of light scattering and diffraction images [16], which allows identification of “signature” features in data such as diffraction images to detect apoptosis [17]. In this report, we present methods for analysis of 3D morphology and nuclear fragmentation of apoptotic human breast cancer MCF-7 cells by confocal imaging and results of comparison to the viable ones.

## Materials and methods

### Cell culture and MTT assay

The human breast carcinoma cell line MCF-7 was purchased from the ATCC and maintained in DMEM medium supplemented with 10% FBS in an incubator with humidified atmosphere of 5% CO_2_ at 37°C. Once MCF-7 cells reached approximately 90% confluence, they were detached with a solution of trypsin/EDTA followed by washing with growth medium. Stock solution of doxorubicin hydrochloride (Sigma, D1515), prepared with deionized water, was added to cell media to induce apoptosis in MCF-7 cells by following established protocols [18–21]. To identify appropriate doses of doxorubicin and treatment times for the current imaging study, we employed the MTT colorimetric assay to determine cell survival curves [22]. For this purpose, detached cells were re-suspended in phenol red-free medium after wash at the concentration of 5x10^5^ cells/ml and then seeded into 96-well plates with 100μl per well. After 24 hours incubation, 100μl of doxorubicin solution were added to obtain a final desired concentration (1~30μM) to treat cells by incubation at 37°C until the time of MTT assay or staining for confocal measurement.

### Fluorescence staining and confocal imaging

Control and treated MCF-7 cells were stained with three fluorescent dyes of Syto-61 (ThermoFisher, S11343), Mito-Tracker Orange (M-7510) and Annexin V (V13241) for confocal imaging. The first two are cell-permeant dyes. The cyanine molecules of Syto-61 can have 40-fold or larger increase in its quantum yield for fluorescence emission once they bind to nucleic acids while affinity of Mito-Tracker Orange is affected by membrane potential of mitochondria. Annexin V, however, binds only to apoptotic cells with phosphatidylserine translocated from the inner to the outer leaflets of the cytoplasmic membrane and thus can be used to detect apoptosis.

Stock solutions of Syto-61 and Mito-Tracker Orange were added to the cell suspension in a centrifuge tube at the concentration of 1μM and 0.2μM, respectively, after harvesting from a 96-well plate. The stained cells were incubated for 30 min before washing with medium by centrifugation. After aspiration of the supernatant, the remaining pellet were suspended in staining buffer, washed and re-suspended in the binding buffer of Annexin V. Annexin V was then added to the solution and the cells were incubate for 15 minutes at room temperature (~24°C) in the dark. Additional binding buffer was added at the end of incubation to the suspension which was placed on ice before imaging.

**Fig 1:**
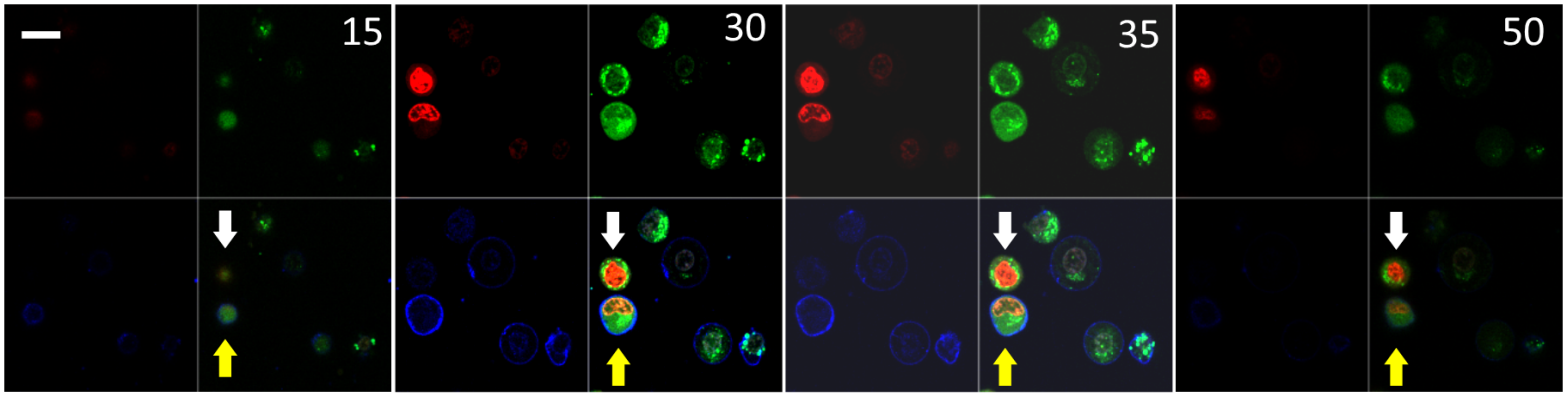
Selected image slices from a stack of 81 slices acquired from MCF-7 cells treated by doxorubicin at a dose of 10μM for 48 hours. Each panel consists of red (upper left), green (upper right), blue (lower left) channels and merged (lower right) with slice number marked. The brightness of blue channels are increased in slices 30 and 35 to make cell membranes stained by Annexin V more visible. This stack contains only 1 viable cell (white arrow) defined by the total blue channel fluorescence less than background value. Bar = 20μm.

For each imaging measurement with a confocal microscope (Zeiss, LSM 700), a suspension sample of triply stained cells with about 140 μl in volume was transferred to a depression glass slide and sealed by a cover glass. To speed up data acquisition with a 63x oil immersion objective, the cell density was kept relatively high so that each field of view (FOV) contained 5 or more cells that can be reconstructed from the same image stack. The fluorescence light signals from Annexin V, Mito-Tracker Orange and Syto-61 binding to their host molecules of specific organelles, upon excitation at 488, 555 and 639nm, respectively, were filtered and saved separately in the blue, green and red channels. Each color image slice consists of 512x512 pixels of 12-bit depth in each color channel. The suspension was translated along the z-axis by steps of 0.5 or 0.6μm in air to acquire a confocal image stack of the FOV. The averaged intensity of Annexin V fluorescence in the blue channel of the acquired image stack on cytoplasmic membrane voxels was used to determine if an imaged cell was viable or apoptotic after 3D reconstruction. Fig 1 displays 4 image slices selected from a stack (see S1 Movie) in which 1 cells was identified as viable and the other 5 cells as apoptotic. One can further see that Syto-61 fluorescent molecules bind much weakly to nucleic acids in apoptotic cells than those in the viable cell while Mito-Tracker Orange exhibit little differences.

### 3D reconstruction

A software named as CIMA (Cell Image and Morphology Analysis) developed on Matlab (MathWorks, 2013a) platform has been significantly improved to analyze MCF-7 cells stained with three fluorescent dyes for this study [7, 23]. We implemented a process of threshold determination for each slice of input image stack based on histogram analysis of the pixel intensity within a user selected region of interest (ROI) for accurate segmentation and reconstruction of intracellular organelles of cytoplasm, mitochondria and nucleus. With individual thresholds obtained for all slices in a stack, morphological operations of dilation, erosion and watershed were combined to divide pixels into different organelle groups in each one of three color channels [24]. For the red channel storing the Syto-61 fluorescence, pixels in each slice are segmented into three groups of background (outside of the cell), cytoplasm and nucleus. The green channel pixels carrying the Mito-Tracker Orange fluorescence are separated into three groups of background, cytoplasm and mitochondria while the blue channel pixels with the Annexin V fluorescence are separated into two groups: on and off cytoplasmic membrane.

Once segmentation completes, the cytoplasmic membrane pixels determined in the red and green channels are compared for each slice and the one of bigger cell area is chosen for reconstruction. A refraction correction factor of 0.87 was determined for the true distance between consecutive image slices from z-axis step size in air [25]. To make voxels as cubic as possible, additional slices are interpolated between acquired consecutive slices followed by voxel based calculation of 3D parameters of nucleus, mitochondria and cell including cytoplasm. The organelles of nucleus and mitochondria for imaging and reconstruction were chosen for their importance in light scattering. In addition to these parameters, the 3D lattice of voxels with organelle index and fluorescence intensity are saved in output data files for further analysis of nuclear and mitochondrial morphology and development of optical cell models [16].

## Results

### Cell survival curve measurement

**Fig 2:**
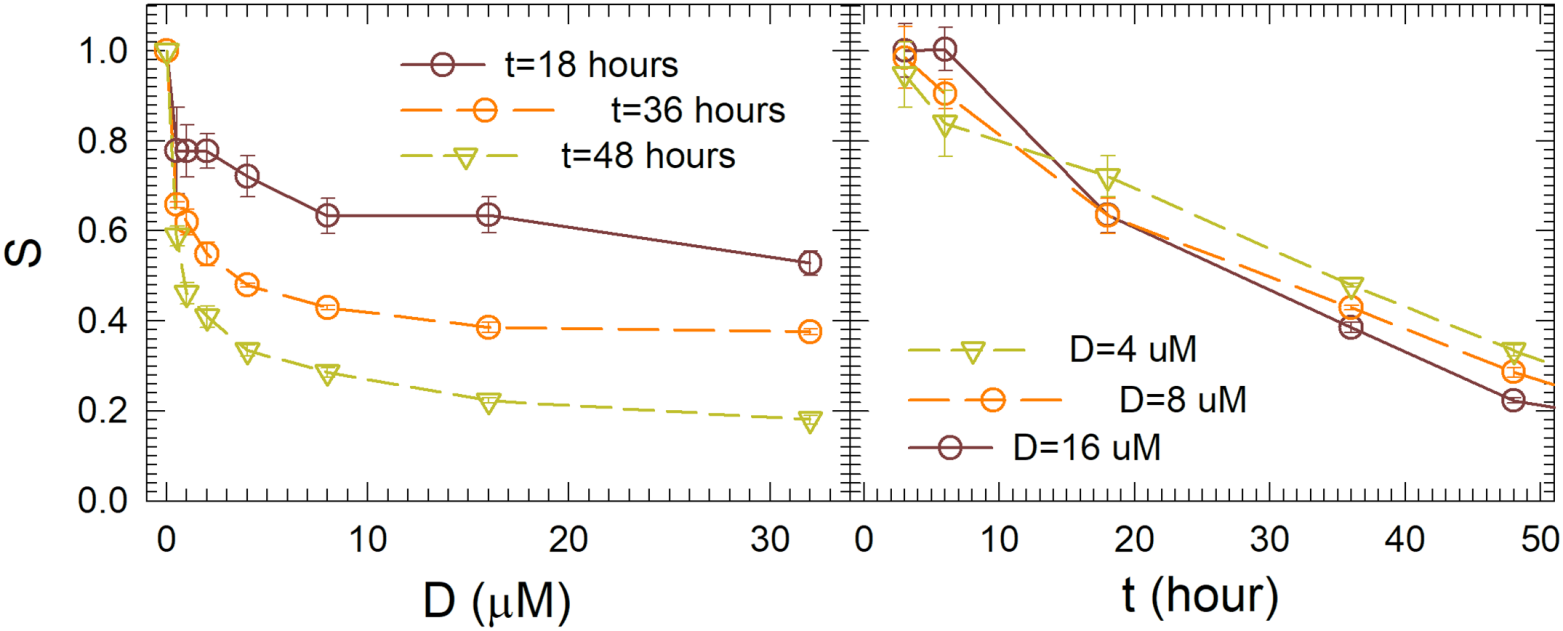
The survival ratios S of MCF-7 cells versus doxorubicin dose D for different treatment time t and versus t for different D. Symbols and error bars represent the means and standard deviations obtained by samples from 8 wells with same D and t. Lines are for guide of eyes.

Cell survival curves were obtained using the MTT assay to confirm established protocols used in this study for ranges of doxorubicin dose D and treatment time t [18–21]. The results are plotted in Fig 2 with symbols and error bars representing respectively the mean values and standard deviations of survival rate S in 8 wells of treated cells against either D or t. The data show that apoptosis in MCF-7 cells induced by doxorubicin is dose dependent for D values up to 10μM and that at 10μM the value of S ranges from about 70% to 30% for t from 18 to 48 hours, respectively. Based on this information, we decided to use doxorubicin concentrations between 8.5 to 10μM and treatment times from 20 to 48 hours to treat MCF-7 cells for confocal imaging.

### Comparison of 3D morphology

Confocal image stacks were acquired from viable and apoptotic MCF-7 cells in 8 suspension samples treated with doxorubicin and survival ratio S ranging from 30% to 70% as stated above and 1 control sample with doxorubicin replaced by growth medium. Reconstruction and analysis of 3D morphology have been conducted on 101 viable and 105 apoptotic cells with the improved CIMA software. Fig 1 presents examples of acquired confocal image slices that include 1 viable and 5 apoptotic MCF-7 cells. Movie files are provided as supporting information for this and another stack (S1 and S2 Movies). A total of 28 morphology parameters were obtained from the reconstructed organelles including cell, nucleus and mitochondria plus 4 parameters of mean value and standard deviation of fluorescence over the voxels of nucleus and mitochondria. To examine the statistical significance of the differences between these parameters of the two cell groups, we obtained p-values by two-tailed student t-test (SPSS, version 24) which are included in Tables 1 to 3. The parameters with p-values less than or equal to 0.05 are regarded as the morphological features with statistically significant differences between the two groups of MCF-7 cells.

**Table 1:** Morphology parameters of MCF-7 cells: Cell ^a^.

Cell parameter	Symbol	Unit	mean ± std ^b^		p-value
			viable (n = 101)	apoptotic (n = 105)	
Grid perimeter	GP_c_	μm	7047±2642	9140±4719	1.17E-04
Surface area	S_c_	μm^2^	1663±448.4	1637±561.4	7.14E-01
Volume	V_c_	μm^3^	3681±1560	3409±1730	2.39E-01
Surface-to-volume ratio	SVr_c_	μm^-1^	0.475±0.0658	0.521±0.121	8.48E-04
Inflection point number	N_si_	-	7.277±6.854	16.57±16.14	2.55E-07
Surface irregularity index	SIi_c_	μm^-1/2^	116.6±27.94	156.7±59.01	3.53E-09
Equivalent radius	ER_c_	μm	9.420±1.217	9.102±1.478	9.41E-02
Volume sphericity index	VSi_c_	-	0.6828±0.0356	0.6592±0.0670	1.83E-03
Mean mem-to-cen distance ^b^	<R_c_>	μm	9.447±1.252	9.257±1.560	3.36E-01
std of R_c_ ^b^	ΔR_c_	μm	0.5275±0.2723	0.7933±0.3876	4.35E-08

^*a*^ Parameters in blue fonts present statistically significant differences between the two groups with n as cell number.

^*b*^ mem-to-cen = membrane-voxels-to-centroid, std = standard deviation.

**Table 2:** Morphology and fluorescence parameters of MCF-7 cells: Nucleus.

Nuclear parameter	Symbol	Unit	mean ± std		p-value
			viable (n = 101)	apoptotic (n = 105)	
Grid perimeter	GP_n_	mm	3520±1504	4566±2739	8.05E-04
Surface area	S_n_	μm^2^	827.8±271.8	820.8±430.5	8.89E-01
Volume	V_n_	μm^3^	1228±580.5	1122±755.1	2.60E-01
Surface-to-volume ratio	SVr_n_	μm^-1^	0.713±0.103	0.798±0.178	3.58E-05
Surface irregularity index	SIi_n_	μm^-1/2^	100.8±29.39	136.4±53.07	1.38E-08
Equivalent radius	ER_n_	μm	6.507±0.9365	6.228±1.151	5.83E-02
Volume sphericity index	VSi_n_	-	0.6612±0.04020	0.6299±0.0672	7.13E-05
Mean mem-to-cen distance	<R_n_>	μm	6.607±1.023	6.558±1.205	7.54E-01
std of R_n_	ΔR_n_	μm	0.9321±0.3569	1.157±0.5314	4.40E-04
Nucleus-to-cell centroid distance	CD_nc_	μm	1.430±0.833	1.718±1.233	5.05E-02
Nucleus-to-cell volume ratio	Vr_nc_	-	0.3396±0.0939	0.3419±0.1316	8.88E-01
Syto-61 fluorescence ^a^	I_n_	-	(15.3±7.08)E04	(9.31±6.46)E04	1.43E-09

^*a*^ Obtained from 12-bit fluorescence intensity of nuclear voxels normalized by incident beam intensity.

**Table 3:** Morphology and fluorescence parameters of MCF-7 cells: Mitochondria.

Mitochondrial parameter	Symbol	Unit	mean ± std		p-value
			viable (n = 101)	apoptotic (n = 105)	
Grid perimeter	GP_m_	μm	313±300	3487±3213	4.08E-01
Surface area	S_m_	μm^2^	773±493	646±468	5.97E-02
Volume	V_m_	μm^3^	251±195	191±163	1.68E-02
Surface-to-volume ratio	SVr_m_	μm^-1^	3.5±0.84	4.1±1.5	3.82E-04
Surface irregularity index	SIi_m_	μm^-1/2^	189±98.2	238±145	4.26E-03
Equivalent radius	ER_m_	μm	0.919±0.236	0.817±0.250	2.82E-03
Mitochondrion-to-cell volume ratio	Vr_mc_	-	0.067±0.038	0.054±0.032	5.99E-03
Mito-Tracker fluorescence ^a^	I_m_	-	(22.0±5.44)E04	(21.2±7.74)E04	4.04E-01

^*a*^ Obtained from 12-bit fluorescence intensity of mitochondria voxels normalized by incident beam intensity.

The first notable results presented by these tables are the statistically significant increases of surface-to-volume ratios and surface irregularity indices for the nuclei and mitochondria of apoptotic cells. Combined with the considerable decrease in mitochondrial volume, these data agree well with the widely known occurrence of nuclear and mitochondrial condensation in apoptotic cells [3]. It is also remarkable that the shapes of cell, nucleus and mitochondria tend to have larger deviations from spherical shapes when cells become apoptotic. Furthermore, Syto-61 fluorescence in nuclei of apoptotic cells present marked reductions in comparison to that of viable cells while the fluorescence of Mito-Tracker Orange in mitochondrial molecules remains about the same. These data are consistent with the previous finding of Syto family stains used as probes for detection of apoptosis due to functionality loss of nucleic acids [26]. Interestingly, visual examination of the fluorescent image stacks indicates that nuclear fragmentation is still relative rare among the apoptotic cells. Most of these cells appear to have intact structures which is confirmed by the fact of no statistically significant differences between surface area and volume for both cell and nucleus as show in Tables 1 and 2. Therefore, the Annexin V positive cells imaged in this study are mostly in the early stage of apoptosis.

### Analysis of nuclear fragmentation

**Fig 3:**
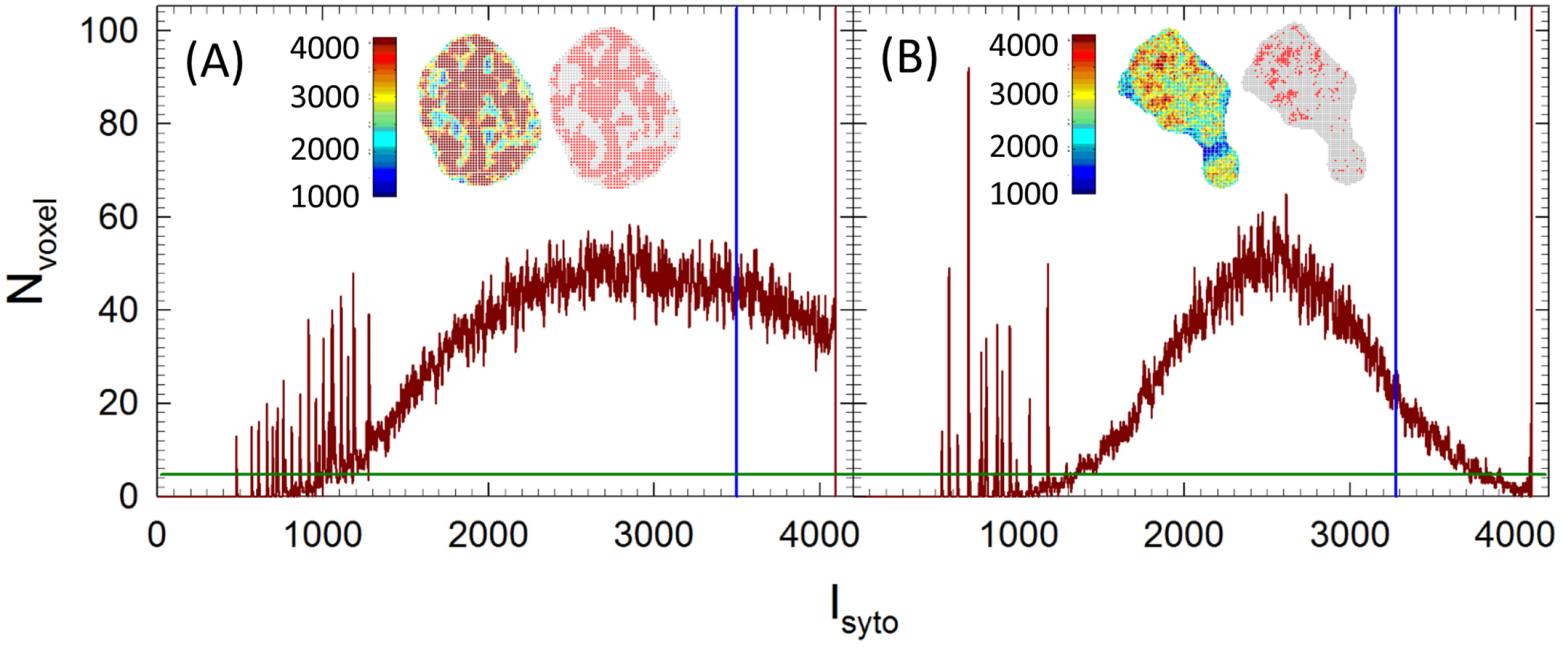
Smoothed nuclear voxel histograms of 12-bit Syto-61 intensity for one (A) viable, (B) apoptotic MCF-7 cell. The dark green line indicates N_5%_ and blue lines indicate I_th_. The insets show the nuclei of two imaged cells extracted from slice #15 with false color for I_syto_ before binary conversion on left and binary image on right with red dots for valid voxels and gray for null ones. The confocal image stacks are shown in supplemental movies.

Nuclear fragmentation is a characteristic event of cell apoptosis [3] and quantitative measurement is highly desired. A new method has been developed to objectively determine fragmentation according to the fluorescence distribution of nuclear stains. The method consists of two steps of binary conversion of fluorescence among nuclear voxels to divide them into two sets of valid and void voxels followed by clustering analysis of valid voxels in the nucleus. Binary conversion of Syto-61 fluorescence is implemented over nuclear voxels after 3D reconstruction. The Syto-61 intensity of all voxels of an imaged cell’s nucleus, I_syto_(**r**), is compiled in a histogram followed by a cubic smoothing over five-point window to reduce fluctuation in I_syto_. A threshold I_th_ is set to the center intensity between I_m_ and I_5%_, where I_m_ is the intensity of maximum voxel number N_voxel_ and I_5%_ is the intensity when N_voxle_ decreases to 5% of the maximum. If N_voxel_ remains higher than 5% of its maximum before I_syto_ becomes saturated at 4095, I_th_ is set to the center intensity between I_m_ and 4095. After determination of I_th_, a nuclear voxel at **r** = (x, y, z) is labeled as either “valid” if I_syto_(**r**) ≥ I_th_ or “null” otherwise. Fig 3 presents two histogram cases to illustrate the determination of I_th_ for binary conversion of Syto-61 fluorescence in nuclear voxels and the insets show the results of binary conversion performed on slice #15 of both image stacks. The acquired confocal image stacks containing the two cells respectively can be seen in the two supplemental files of S1 and S2 Movies.

To quantify nuclear fragmentation, we set out to analyze the spatial distribution of valid nuclear voxels in 3D space of **r** = (x, y, z) by examination of voxel density in a given volume. The conventional k-means algorithm, as implemented by the function of “kmeans” in Matlab, has been widely used to automatically determine the “closeness” among elements of a set in a parameter space and optimal arrangement of the elements in k clusters. The algorithm, however, requires *a priori* knowledge on k. For determination of an optimized k for a given nucleus, we developed a density based dividing scheme that is easy to implement and effective for our study in comparison to other schemes [27]. It starts with k = 1 or the assumption that all valid nuclear voxels belong to one cluster and calculates the centroid. Euclidean distances of these voxels in the space of **r** to the centroid are then calculated. A sphere centered at the centroid with radius equal to the distance of the furthest valid voxel from the centroid is used to obtain density ρ_11_, which is defined as the number ratio of valid nuclear voxels to all voxels enclosed by the sphere. The k is then incremented to 2 by dividing all valid voxels into two daughter clusters of 21 and 22 followed by minimizing their Euclidean distances to each centroid and calculation of ρ_21_ and ρ_22_ for the two clusters. The densities are then checked by the following inequality

$$\frac{1}{2}(\rho_{21}+\rho_{22})\geq 1.5\rho_{11}.$$ (1)

The cluster division is kept if the above is true or the average density of two daughter clusters becomes significantly larger than that of parent cluster. Otherwise the division is abandoned and all valid voxels remain in the parent cluster. The division process repeats until the inequality Eq (1) becomes false for each pair of daughter clusters.

**Fig 4:**
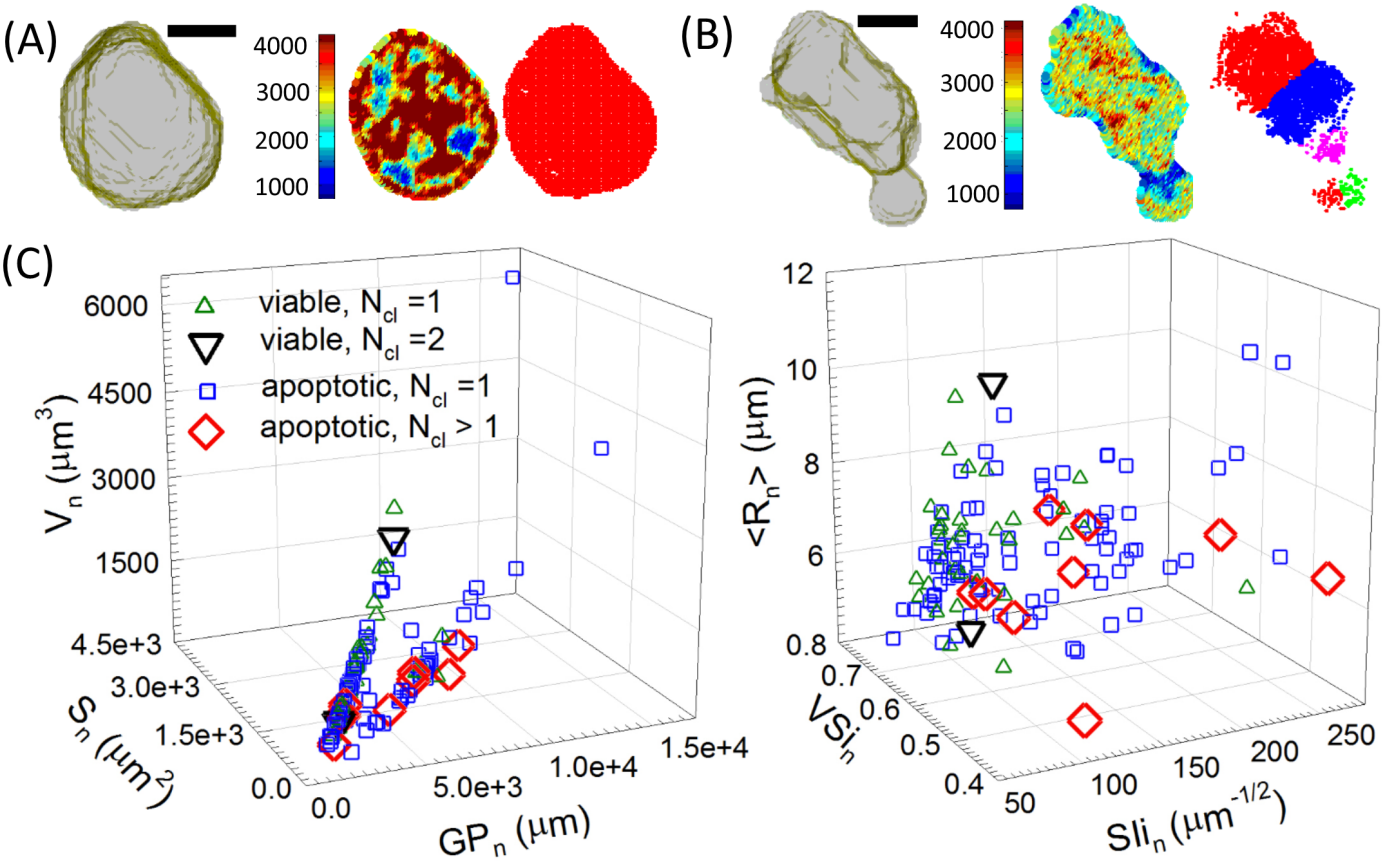
Two cases of clustering analysis on nuclear voxels of one (A) viable MCF-7 cell with N_cl_ = 1 (see Visualization 1); (B) apoptotic one with N_cl_ = 5 (see Visualization 2): 3D perspective view of nucleus (left), false-color image slice #18 before (middle) and after (right) binary conversion, bar = 5μm; (C) scatter plots of nuclear parameters of cells that allow voxel clustering: V = volume, S = surface area, GP =(number of nuclear membrane voxels)·(voxel side length), SIi = GP/V^1/2^, VSi = 4πR^2^/S with R = (3V/4π)^1/3^.

The above clustering analysis relies on the existence of a peak value in the smoothed fluorescence histogram of nuclear voxels. Usually this should always be the case if the FOV of confocal imaging contains only one or two cells which allows adjustment of incident beam intensity and/or exposure time to avoid severe underexposure or overexposure on imaged cells. In this study we included larger number of cells in FOV to reduce data acquisition time and its effect on cells’ viability. The imaged cells and their nuclei have large variations in affinity to Syto-61 dye. As a result, it is very difficult to select appropriate imaging parameters so that all cells in FOV have peaked histograms for clustering nuclear voxels. Among the 101 viable cells with 3D reconstruction performed, 35 cells have Syto-61 fluorescence histograms that permit clustering calculations while 83 out of 105 apoptotic cells allow the same calculations. Fig 4 presents two cases of nuclear voxel cluster analysis of MCF-7 cells and the scatter plots of nuclear parameters of the 118 cells whose numbers of clusters can be calculated as N_cl_.

Out of the 35 viable cells plotted in Fig 4(C), only 2 have their cluster numbers N_cl_ of nuclear voxels larger than 1 and both are at 2. In comparison, 9 out of 83 apoptotic cells have their N_cl_ ranging from 2 to 5 and Fig 4(B) illustrates the only case of N_cl_ = 5. The plots in Fig 4(C) present clear evidences that the apoptotic MCF-7 cells have significantly larger variations than those of viable cells, which are particularly true in surface shape related parameters as shown in the right plot of Fig 4(C). Combined with the visual examination of the confocal images and data in Tables 1 to 3, it becomes apparent that nearly all of the apoptotic cells imaged and analyzed in this study were in the early stage of apoptosis in which nuclear fragmentation is rare but can be quantified using the clustering analysis method. Hence the new method provides an objective tool for detection of apoptosis in early stage and quantification of nuclear fragmentation.

## Discussion and summary

The 3D morphological parameters of apoptotic and viable MCF-7 cells have been obtained and analyzed through this study with a significantly improved image segmentation and 3D reconstruction software. Automated quantification of nuclear fragmentation has been achieved with a new method for clustering analysis of nuclear voxels in a 3D reconstructed cell according to their fluorescence distribution. Nearly all apoptotic cells as identified by the Annexin V stained cytoplasm membrane in confocal images were found to be in the early stage with statistically similar surface areas of cell, nuclei and mitochondria and volumes of the first two organelles. Despite the fact that they are in early stage, the apoptotic cells and their nuclei show significant increase in deviation of surface shapes from spherical ones or degree of irregularity. Loss of Syto-61 fluorescence in nuclei of these cells can also be observed in confocal image stacks and with the data of Table 2 that is likely caused by oligonucleosomal DNA fragmentation [26]. With the k-means clustering based method, we determined the number of clusters of voxels for objective evaluation of nuclear fragmentation, which occurs only in 9 of 83 apoptotic cells imaged and analyzed in this study. One thus can conclude that the increased irregularity of cytoplasm and nuclear surfaces and molecular changes in nucleic acids likely precede the occurrence of nuclear fragmentation and could serve as the markers for early detection of apoptosis. These results provide very useful insights on detection of apoptosis, particularly in the early stage, by polarized and coherent light scattering which is caused by induced oscillations of molecular dipoles in single cells. Utilization of 3D morphology data acquired through this study is underway to develop accurate optical models of viable and apoptotic MF-7 cells and identify pattern features in diffraction images by the p-DIFC method.

In summary, we have performed confocal imaging and 3D morphology analysis on 206 viable and apoptotic MCF-7 cells induced by doxorubicin and triply stained by Annexin V, Syto-61 and Mito-Tracker Orange fluorescent dyes. With a new method for clustering analysis of nuclear voxels, it has been shown that the apoptotic cells in early stage exhibit significantly increases in shape and molecular changes of cytoplasm, nucleus and mitochondria. These results deepen notably our understanding of early apoptosis in terms of 3D morphology and nuclear fragmentation, which allows future development of morphology-sensitive and label-free tools for apoptosis detection by, for example, diffraction imaging.

## Supporting information

S1 Movie A video file of displaying slices of a confocal image stack acquired from MCF-7 cells treated by doxorubicin at a dose of 10μM for 48 hours. The cells were triply stained with Annexin V, Mito-Tracker Orange and Syto-61. This stack contains 5 apoptotic cells and 1 viable cell, which can be seen above the center line on the left side of the field-of-view and is absent of the fluorescence of Annexin V recorded in the blue channel of the color data file. The slice numbers are indicated at the lower-right corner.
(MP4)

S2 Movie A video file of displaying slices of a confocal image stack acquired from MCF-7 cells treated by doxorubicin at a dose of 8.5μM for 24 hours. The cells were triply stained with Annexin V, Mito-Tracker Orange and Syto-61. This stack shows one late-stage apoptotic cell above the center line to the right of the middle in the field-of-view that has fragmented nucleus and shrinking cytoplasm. The slice numbers are indicated at the lower-right corner.
(MP4)
